# SARS-CoV-2 investigation in cerebrospinal fluid from meningitis patients during the first pandemic wave

**DOI:** 10.1016/j.bjid.2025.104571

**Published:** 2025-08-05

**Authors:** Maria Cecilia Cergole-Novella, Thayná Rosa Bispo, Elaine Monteiro Matsuda, Daniela Rodrigues Colpas, Ivana Barros de Campos

**Affiliations:** a Instituto Adolfo Lutz, Centro de Laboratório Regional Santo André, Santo André, SP, Brazil; b Prefeitura Municipal de Santo André, Secretaria de Saúde de Santo André, Santo André, SP, Brazil

**Keywords:** SARS-CoV-2, RT-qPCR, Meningitis, Cerebrospinal fluid

## Abstract

SARS-CoV-2 predominantly affects the respiratory system; however, during the first pandemic wave, there was a concern about its neuroinvasive potential due to its ability to replicate in neural cells and the neurological signs widely reported as impaired taste or smell. This study aimed to evaluate the presence of SARS-CoV-2 in cerebrospinal fluid (CSF) from suspected cases of meningitis. During the first year of the pandemic, from January 2020 to February 2021, CSF samples were first submitted to multiplex qPCR to detect the three main bacteria causing meningitis (*Neisseria meningitidis, Streptococcus pneumoniae*, and *Haemophilus influenzae*), as the routine of the laboratory, but also, they were submitted to RT-qPCR to SARS-CoV-2 detection. Some samples (20 %) were positive for one bacterium; however, none were positive for the virus, suggesting that the incidence of SARS-CoV-2 in meninges is extremely low. This study with other data in the literature contributes to the epidemiologic surveillance of SARS-CoV-2.

Meningitis is a serious neurological manifestation characterized by inflammation of the meninges with high rates of mortality and morbidity worldwide. Examination of cerebrospinal fluid (CSF) is of paramount importance for the diagnosis of all forms of meningitis although significant overlap in the CSF profile among pathogens exists.^1^ The early identification of the etiological agent is important for patient care and epidemiologic surveillance purposes. The COVID-19 pandemic surprised the world due to its diverse presentation and complexity, not restricted to a respiratory disease with multisystem complications, including acute and chronic neurological manifestations, even in cases without respiratory symptoms.^2^ Some studies have shown the association of SARS-CoV-2 present in the nasopharyngeal samples of hospitalized patients with compromised neurological functions during the first pandemic wave.^3^^,^^4^ However, the studies related to the presence of SARS-CoV-2 in CSF samples showed that only a few cases have been tested.^5^ In a systematic review of cases report, SARS-CoV-2 was detected in only four of 21 CSF samples of patients with neurological manifestations by the real-time Reverse-Transcriptionquantitative Polymerase Chain Reaction (RT-qPCR).^6^ Others reported the first case of SARS-CoV-2-associated to meningitis/encephalitis that was not detected in the nasopharyngeal swab but was detected in the CSF sample.^7^ Further research and longer follow-up are required for more conclusive results because SARS-CoV-2 is related to encephalitis/meningitis, but it has not been established if there are specific clinical characteristics of encephalitis/meningitis associated with SARS-CoV-2 infection.^8^ Accordingly, the present study aimed to evaluate the presence of SARS-CoV-2 in CSF samples from suspected cases of meningitis that were received in a health public laboratory for routine evaluation, during the first year of the COVID-19 pandemic and before vaccination.

CSF samples from patients classified as suspected cases of meningitis were first submitted to cytological, biochemical, culture, and Gram stain assays in tertiary diagnostic laboratories. Then, an aliquot was sent to our health public laboratory of the region to confirm the diagnosis of meningitis and to provide information for epidemiological surveillance, from January 2020 to February 2021. As part of the laboratory routine, it was performed the multiplex real-time PCR (qPCR) for bacterial meningitis diagnosis. DNA extraction was performed using 500 µL of samples and the PureLink™ Genomic DNA Mini Kit (Thermo Fisher Scientific, USA). The multiplex qPCR assay was performed as previously described and modified^9^^,^^10^ to detect *Neisseria meningitidis, Streptococcus pneumoniae,* and *Haemophilus influenzae*, which are the leading causes of bacterial meningitis worldwide. The same CSF samples were also submitted to a chemical/heating extraction procedure to obtain RNA using QuickExtract DNA Extraction Solution (Lucigen, USA). After that, RT-qPCR was performed using the Allplex™ 2019-nCoV Assay Kit (Seegene, South Korea) to identify the presence of E, N, and RdRP genes from SARS-CoV-2. Samples were considered positive with a cycle threshold of up to 40 for at least one of the exclusive SARS-CoV-2 genes (N or RdRP), according to the manufacturer's instructions. The RT-qPCR was also performed to evaluate the presence of the human RNase P gene (RP), as nucleic acid extraction control.^11^ All PCR techniques were performed in duplicate.

Fifty-three CSF samples were received during the first year of the pandemic. The qPCR was performed for bacterial meningitis diagnosis for all samples. Due to the lack of enough material to perform all assays, 50 CSF samples were tested for SARS-CoV-2 detection. Table 1 presents the cytological, biochemical, culture, and other data from these samples. Only a few samples were positive for bacteria in culture or bacterioscopy (five and ten samples, respectively) in the tertiary laboratories.

**Table 1:** Data from suspected cases of meningitis from January 2020 to February 2021.

	SARS-CoV-2 tested
**N**	50
**Age (mean)**	32.3 (IQR 14.05‒54.7)
**Sex (n)**	
Male	32 (64 %)
Female	18 (36 %)
**Race (n)**	
White	22 (44 %)
Brown	11 (22 %)
Black	2 (4 %)
Yellow	1 (2 %)
Missing information	14 (28 %)
**Comorbidities (n)**	13 (26 %)
Not informed	10 (20 %)
**Death (n)**	3 (6 %)
**Neurological symptoms (n)**	
Fever	27 (54 %)
Headache	22 (44 %)
Vomit, nausea	21 (42 %)
Neck stiffness	10 (20 %)
Mental confusion	8 (16 %)
Convulsion	8 (16 %)
Others	36 (72 %)
Not informed	5 (10 %)
Median time from symptoms to sample collection (days)	3 (IQR 1‒7)
**External laboratory data**	
Positive culture	5 (10 %)
Negative culture	20 (40 %)
Culture not performed	4 (8 %)
Culture not informed	21 (42 %)
Positive bacterioscopy	10 (20 %)
Negative bacterioscopy	35 (70 %)
Bacterioscopy not informed	5 (10 %)
Leukocytes count (cells/mm^3^)	1305 (IQR 109‒4048)
Glucose (mg/dL)	38 (IQR 13‒53)
Protein (mg/dL)	158 (IQR 88‒230.5)
Median time from sample collection to our laboratory receiving (days)	5 (IQR 2‒10)
**Multiplex qPCR (n)**	
*Neisseria meningitidis*	4 (8 %)
*Streptococcus pneumoniae*	6 (12 %)
*Haemophilus influenzae*	0
Negative	40 (80 %)

Categorical variables are presented as the number of cases and proportion. Continuous variables are expressed as the median and the interquartile range (IQR 25-75 %).

Ten of the 50 samples (20 %) were positive for one bacterium evaluated by the multiplex qPCR. Six were diagnosed with *Streptococcus pneumoniae*, four with *Neisseria meningitidis*, and none were positive for *Haemophilus influenzae*. When the multiplex qPCR results were compared to the culture and bacterioscopy performed by the tertiary laboratories, only one positive culture agreed with the qPCR, and four positive results in bacterioscopy were equivalent to the qPCR. A total of 16 samples were negative in the culture and the qPCR. Only two samples presented the culture and the bacterioscopy assay in agreement and they were positive for other bacteria not evaluated by the qPCR. From the three deaths observed in this group, none were qPCR positive.

None of the 50 CSF samples were positive for either E, N, or RdRP genes by the RT-qPCR for SARS-CoV-2 (Fig. 1). Moreover, the same samples were tested in the RT-qPCR for the human RP gene, and the success of extraction was confirmed. The closure of the cases was that 24 out of 50 cases (48 %) remained as undetermined meningitis when there was no definition of the etiological agent, of which 8 presented cytological and biochemical results according to viral meningitis, and 16 were not possible to conclude the type of meningitis based on all results. Twenty-six cases (52 %) were concluded as bacterial meningitis, of which 16 had determined the bacteria, and 10 were concluded as undetermined bacterial meningitis based on cytological and biochemical results since specific assays produced negative results. Coinfection with SARS-CoV-2 was not observed in all bacterial meningitis cases. Moreover, searching for other analyses in the database of the laboratory, from all 53 patients, three had nasopharyngeal swabs collected due to respiratory symptoms on the same day of CSF sample collection, or less than a month before the meningitis episode. Of these, two were positive for SARS-CoV-2 in the nasopharynx. One of the nasopharyngeal-positive patients was negative for SARS-CoV-2 in the CSF sample. The other was one of the patients who died, and there was no sufficient volume for the RT-qPCR.

**Fig. 1:**
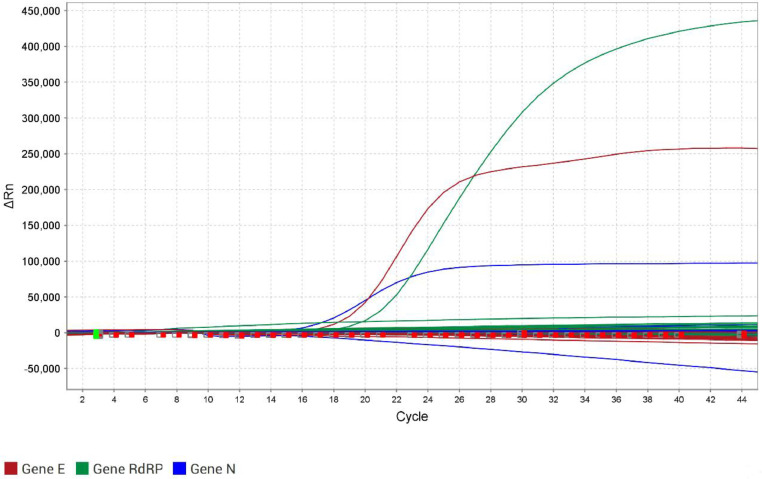
Amplification curves of the three genes from the RT‐qPCR for SARS-CoV-2 detection, using the Allplex™ 2019-nCoV Assay Kit (Seegene, South Korea) in the QuantStudio™ 5 Real-Time PCR System (Applied Biosystems™, Thermo Fisher Scientific). The curves are: red for the E gene, blue for the N gene, and green for the RdRP gene. The y‐axis shows ΔRn, which is the magnitude of the normalized fluorescence signal generated by the fluorophore at each cycle during the PCR amplification. The x‐axis shows the number of cycles. Only the positive control crossed the threshold of 8000 ΔRn for the E gene, 13,000 for the N gene, and 25,000 for the RdRP gene.

This study evaluated the ability of SARS-CoV-2 to infect the meninges, which was highly concerned about its neuroinvasive potential at the beginning of the SARS-CoV-2 pandemic. It applied an easy and inexpensive methodology for RNA extraction. Although this procedure does not concentrate on the genetic material, as standard extraction methodologies, like silica column or magnetic beads, thus it is expected to have lower sensitivity than the other methodologies and potentially lead to false negative results. On the other hand, the Lucigen kit allows the extraction of low volumes which could mitigate the issue of scarce CSF sample volumes, and using the RP gene as the positive control could indicate that the extraction was performed properly and has enough RNA material for RT-qPCR. Usually, there is not enough volume to be applied in the standard extraction protocols; also, another point in favor is that the manufacturer claims the kit disintegrates inhibitory compounds for PCR amplification.

This study has many limitations, such as the small number of CSF samples tested for SARS-CoV-2, which limits the statistical power and does not allow generalization of the results. Despite that, this small number of samples represents all suspected cases of bacterial meningitis, which were confirmed or unconfirmed, in the first year of the pandemic, in a population of 2.5 million from seven cities in the Sao Paulo metropolitan area, Brazil. This amount was less than half of the usual number of assays to diagnose bacterial meningitis previously observed in the region (an annual average of 230 qPCR assays between 2010 to 2013 and 130 assays from 2014 to 2018).^12^ The decrease observed in our study was probably due to the containment measures of COVID-19, such as lockdown, mask-wearing, and social distancing implemented during this period (years 2020‒2021). This reduction of invasive diseases caused by the main three bacteria also happened in many countries where these measures were applied since they also contribute to diminishing the transmission of other respiratory pathogens that cause meningitis.^13^ As far as we are aware, there was no notification of meningitis cases caused by SARS-CoV-2 in the region of this study accordingly to our results. However, this absence of SARS-CoV-2 in CSF samples may reflect the regional epidemiology rather than the virus behavior in humans; thus, these results may not be applicable beyond the local population and the period of time of this study. There was only one case reported of COVID-19-associated meningoencephalitis in our country, which happened in the first year of the pandemic.^14^ Another drawback is that only suspected cases of meningitis were evaluated in this study, thus patients were not selected for other neurological symptoms suggestive of viral Central Nervous System (CNS) disease, which may underestimate the SARS-CoV-2 association.

The findings obtained in this study suggest that SARS-CoV-2 has a low predilection to infect the meninges, which corroborates Destras et al.^15^ They observed only two CSF samples positive for SARS-CoV-2 in a larger cohort, but they suspected it was cross-contamination with blood.^15^ The COVID-19-related neurological manifestations are still under debate. Some argue that these symptoms result from the direct SARS-CoV-2 invasion and infection of the central nervous system (CNS) and some attribute the CNS sequelae as a result of systemic inflammatory responses triggered in the periphery.^16^^,^^17^^,^^18^ This study adds to this discussion by suggesting that systemic inflammation could cause neurological manifestations, and thus it could be the reason the virus was not detected in the CSF samples. However, this study is retrospective and observational, restricting causal inferences or analysis of other factors.

This study had other limitations, as it was carried out in cases without an etiological diagnosis, and other viruses typically causing meningitis/encephalitis (e.g. Herpes Simplex Virus or Enterovirus) were not examined, limiting the differential diagnosis and broad pathogen estimation. More investigative studies like ours can increase the knowledge base, even with a small number of samples.

## Data availability

All data generated or analyzed during this study are included in this published article.

## Ethics approval

The study was approved by the Institutional Ethical Committee (CAAE: 45285921.7.0000.0059), and waived the informed consent from patients for this study since clinical specimens reported here were collected for diagnostical purposes and data were analyzed anonymously.

## Authors’ contributions

Conception and design of the study: Ivana Barros de Campos; Material preparation and data collection: Thayná Rosa Bispo, Daniela Rodrigues Colpas; Formal analysis and investigation: Thayná Rosa Bispo, Daniela Rodrigues Colpas, Maria Cecilia Cergole-Novella, Elaine Monteiro Matsuda, Ivana Barros de Campos; Resources: Ivana Barros de Campos; Funding acquisition: Ivana Barros de Campos, Maria Cecilia Cergole-Novella; Writing-original draft preparation: Ivana Barros de Campos, Elaine Monteiro Matsuda, Maria Cecilia Cergole-Novella; Writing-review and editing: Ivana Barros de Campos, Elaine Monteiro Matsuda, Maria Cecilia Cergole-Novella; Supervision: Ivana Barros de Campos. All authors have read and approved the final manuscript.

## Funding

This work was partially supported by Fundação de Amparo à Pesquisa do Estado de São Paulo (FAPESP): grants n° 2021/01194–0 (TRB ‒ 2021), 2017/03022–6 (IBC – 2017‒2021), and 2020/16662-6 (MCCN – 2023-2025).

## Conflicts of interest

The authors declare no conflicts of interest.
